# Interrelationships between urban travel demand and electricity consumption: a deep learning approach

**DOI:** 10.1038/s41598-023-33133-y

**Published:** 2023-04-17

**Authors:** Ali Movahedi, Amir Bahador Parsa, Anton Rozhkov, Dongwoo Lee, Abolfazl Kouros Mohammadian, Sybil Derrible

**Affiliations:** 1 Department of Civil, Materials, and Environmental Engineering, University of Illinois at Chicago, 842 W Taylor Street (M/C 246), Chicago, IL 60607 USA; 2 Department of Urban Planning and Policy, University of Illinois at Chicago, 412 S Peoria St, Chicago, IL 60607 USA; 3 Department of Policy and Administration, Incheon National University, Incheon, 22012 South Korea; 4 Institute for Environmental Science and Policy, University of Illinois at Chicago, 1603 West Taylor Street, Chicago, IL 60607 USA

**Keywords:** Civil engineering, Computer science

## Abstract

The analysis of infrastructure use data in relation to other components of the infrastructure can help better understand the interrelationships between infrastructures to eventually enhance their sustainability and resilience. In this study, we focus on electricity consumption and travel demand. In short, the premise is that when people are in buildings consuming electricity, they are not generating traffic on roads, and vice versa, hence the presence of interrelationships. We use Long Short Term Memory (LSTM) networks to model electricity consumption patterns of zip codes based on the traffic volume of the same zip code and nearby zip codes. For this, we merge two datasets for November 2017 in Chicago: (1) aggregated electricity use data in 30-min intervals within the city of Chicago and (2) traffic volume data captured on the Chicago expressway network. Four analyses are conducted to identify interrelationships: (a) correlation between two time series, (b) temporal relationships, (c) spatial relationships, and (d) prediction of electricity consumption based on the total traffic volume. Overall, from over 250 models, we identify and discuss complex interrelationships between travel demand and electricity consumption. We also analyze and discuss how and why model performance varies across Chicago.

## Introduction

The analysis of infrastructure use data in relation to other components of the infrastructure can help better understand interdependencies and interrelationships between them, with the potential to enhance their sustainability and resilience. Indeed, no infrastructure system works in isolation. All infrastructure systems—including transport, water, wastewater, electricity, gas, and telecommunications—are interdependent^1,2^. In part because of these interdependencies, but also intrinsic to how people live, the way infrastructure systems are used is also interrelated. For example, Movahedi and Derrible^3^ showed that electricity, gas, and water consumption in large-scale buildings are interrelated (i.e., the consumption of one can be predicted by the two others). Zhang and Qian^4^ classified the patterns of electricity consumption over a night to estimate the traffic congestion of a highway in the morning. Overall, infrastructure systems are often more interrelated than initially expected, for example by sharing physical surface and subsurface space^5^ and by competing for time and resources^6,7^.

In this study, by using zip code-level electricity data as well as traffic loop detector data, we seek to identify and understand interrelationships between travel demand and electricity demand. More precisely, using traffic data to count the number of vehicles entering and exiting a zip code can capture the number of people in a zip code at a given time who may be in buildings otherwise, consuming electricity. Concurrently, a decrease in electricity consumption can express that people have left a building and may use a vehicle, generating traffic. In this study, we use electricity consumption data of several zip codes in Chicago at 30-min intervals for November 2017. To achieve our goal, we use Long Short Term Memory (LSTM) network—a type of deep learning model—to model electricity consumption patterns of zip codes based on the traffic volume of the same zip code and nearby zip codes. The specific objectives of the study are to:

- Understand the correlation between electricity consumption and traffic volume.
- Investigate the temporal relationships between electricity consumption and traffic volume.
- Investigate the spatial relationships between electricity consumption and traffic volume.
- Develop models to predict electricity consumption based on traffic volume.

In the next section, we review the literature on electricity consumption and traffic modeling, and on interrelationships between infrastructure systems in cities. After, we describe the electricity consumption and traffic datasets used in the study. Next, we go over the results by addressing each objective sequentially, and we then discuss these results. Finally, we explain in detail the methodological approach utilized in the study.

## Literature review

The electricity power grid is a complex system with many components^8^. The stable and uninterrupted operation of the power grid plays a vital role in economic development, national security, and overall social welfare. As of this writing, electricity cannot be cheaply and effectively stored in required massive amounts. As a result, electric utilities and other power market players must forecast electricity consumption in the (a) short-term (few minutes to hours), (b) mid-term (hours to a day ahead), and (c) long-term (seasonal/annual, up to a few years) in generation, transmission, and distribution networks. Thanks to the deployment of smart meters, predictions have become generally more accurate. This accurate forecasting of electricity consumption levels is crucial for power systems, and the selected method for making predictions provides a better understanding of the dynamics of the system and can even help ease operating costs for market players. The traditional predictive techniques include the construction of mathematical and statistical models such as auto-regressive and moving average (ARMA) models^9^; auto-regressive integrated moving average (ARIMA) models^10^; multiple linear regression (MLR) and principal component analysis (PCA) models^11^; gray models (GM)^12^; and Kalman filter-based (KF) models^13^. Nonetheless, traditional statistical models are known to be limited. For instance, GM models are not always effective for electrical load forecasting but work better for addressing small sample problems^14^ and ARMA models may fail to consider the influence of random variables other than in typical time series forecasting methods^10,14^. This means that traditional statistical models work well in normal daily conditions, but they become less reliable while dealing with meteorological, sociological, and economic changes^15^ or with relations to other systems.

To deal with complex nonlinear relationships, machine learning (ML) and deep learning techniques are generally preferred. The following techniques are mentioned in the literature: artificial neural networks (ANN)^16–18^ fuzzy-logic-based algorithms^19,20^, genetic-algorithm-based (GA) neural network^21^, support vector machine (SVM)^22^, tree-based models^23–25^, LSTM-based neural network^26^; single hidden layer network configurations with random weights (RWSLFN)^27^, and multilayer perceptron (MLP)^28^ to name a few. In the literature, LSTM has been shown to perform particularly well on time series data for a range of applications, including to predict the spread of COVID-19^29–32^. Specifically looking at traffic forecasting and flow prediction, several studies^33,34^ also found that LSTM performed better than traditional techniques like ARIMA or other ML techniques like support vector regression (SVR). In this study, we have opted to solely use LSTM as our main goal is not to find the best performing model but to investigate the presence of interrelationships between electricity consumption and traffic volume.

As many studies demonstrate^35–51^, electricity consumption is linked to myriads of variables, from urban characteristics (e.g., morphology, density) and building characteristics (e.g., size and insulation technology) to weather characteristics (e.g., temperature and cloud coverage) and socio-economic and demographic characteristics (e.g., household income and age). Yet, this list is not exhaustive. As infrastructure systems are interdependent and interrelated by nature^52^, electricity consumption is also linked to demand patterns for other infrastructure services, such as residents commute time, traffic, and urban mobility patterns, suggesting that traffic network data can be used as a source of information to predict electricity consumption as well^53^. To date, little research has been carried out and not many studies are available that focus on the interrelation between travel and electricity demand. Few studies explore the causal interdependencies between electricity, transport, and weather data^53,54^. Gilanifar et al.^55^ developed a Bayesian Gaussian Process model that explores usage of electricity to enhance short-term load forecasting. Aparicio et al.^56^ studied the dependencies between power demand and road traffic data using linear correlation and compare the results with other standard features, such as historical load and temperature.

## Data

### Electricity

In this article, we work with anonymized energy usage data in 30-min intervals at the zip code level within the city of Chicago collected by the local utility Commonwealth Edison (ComEd) and accessible (for a fee)^57^. Each measurement in the dataset represents the total electricity consumed (in kWh) for a specific customer in a certain time interval (30 min). We decided to build our research on this dataset because we assume that the raw high-resolution interval data that we get directly from the automated metering infrastructure (AMI) have a high level of accuracy and fidelity.

Interval data from AMI has become widely available to utilities throughout the U.S.^58^. It is often used to identify energy use trends and peaks in the interest of anomaly detection^59^ and to make predictions of electricity consumption^60^ to improve the stability of the power grid. Household data include load shapes measured at the household level considers seasonal and daily fluctuations and show significant differences in electricity consumption during the day, week, month, and year. We are interested in observing the loads in one month with a specific focus on the time of the day and the day of the week. We used residential electricity consumption from 28 zip codes located along the main transport corridors of Chicago: I-290, I-90, I-55, I-57, and I-94 interstate expressways for the month of November 2017.

While beneficial for both utilities and customers, data collected and utilized using AMI systems have caused concerns regarding customers’ privacy^61^. Although Martínez et al.^62^ observes a potential privacy issue of simple anonymization methods, the distribution of fine-grained data is normally considered acceptable as long as they cannot be linked to the households they originate from through an anonymization process^63^.

In this study, we use data that consists of fine-grained records of electricity consumption aggregated by 5-digit zip codes where specific identifiers, including but not limited to name, address, and electric account number, are omitted. Table 1 shows average electricity consumption per building in each zip code (in kWh). The table also includes area (in square kilometers) and population (based in American Community Survey (ACS) 2019 5-Year Data) for each zip code for the interest of the reader.

**Table 1:** Average electricity consumption (kWh).

Zip code	12 AM to 3 AM	3 AM to 6 AM	6 AM to 9 AM	9 AM to 12 PM	12 PM to 3 PM	3 PM to 6 PM	6 PM to 9 PM	9 PM to 12 AM	Area (km^2^)	Population
60607	1.09	1.07	1.30	1.44	1.45	1.43	1.41	1.27	6.06	30,306
60608	0.57	0.56	0.66	0.71	0.71	0.70	0.71	0.65	16.78	80,011
60609	0.71	0.70	0.84	0.91	0.91	0.88	0.88	0.81	20.18	60,551
60612	0.69	0.68	0.83	0.92	0.91	0.88	0.85	0.79	9.63	32,240
60614	0.52	0.50	0.60	0.65	0.66	0.69	0.75	0.66	10.00	72,391
60616	0.53	0.51	0.58	0.63	0.63	0.64	0.67	0.62	11.58	52,557
60618	0.40	0.38	0.45	0.50	0.51	0.53	0.57	0.50	13.11	94,646
60619	0.39	0.37	0.40	0.42	0.42	0.43	0.48	0.45	15.85	61,372
60620	0.44	0.42	0.46	0.48	0.48	0.49	0.54	0.51	18.13	68,761
60621	0.47	0.43	0.47	0.50	0.49	0.50	0.53	0.50	9.71	26,736
60623	0.41	0.39	0.44	0.48	0.49	0.49	0.52	0.48	14.14	77,732
60624	0.50	0.48	0.55	0.61	0.60	0.60	0.61	0.56	9.19	35,054
60625	0.30	0.28	0.33	0.36	0.37	0.39	0.43	0.39	9.84	78,820
60628	0.58	0.56	0.61	0.64	0.63	0.64	0.68	0.66	28.62	65,008
60630	0.36	0.34	0.40	0.44	0.45	0.47	0.51	0.45	12.28	55,692
60631	0.42	0.40	0.49	0.56	0.56	0.59	0.63	0.55	9.71	28,864
60632	0.59	0.58	0.73	0.81	0.81	0.79	0.79	0.71	19.42	86,715
60633	0.57	0.56	0.64	0.66	0.64	0.64	0.69	0.65	30.10	12,720
60637	0.37	0.35	0.38	0.39	0.39	0.40	0.45	0.42	11.97	46,621
60641	0.36	0.34	0.41	0.45	0.46	0.47	0.51	0.45	10.44	70,163
60642	0.31	0.29	0.34	0.38	0.41	0.42	0.46	0.41	4.53	20,191
60643	0.50	0.47	0.53	0.57	0.56	0.60	0.66	0.60	19.09	48,572
60644	0.44	0.42	0.47	0.51	0.50	0.50	0.53	0.50	9.12	45,919
60647	0.37	0.34	0.40	0.44	0.45	0.47	0.51	0.53	10.33	85,658
60656	0.34	0.32	0.38	0.41	0.42	0.44	0.49	0.44	8.39	28,982
60661	0.95	0.94	1.16	1.31	1.31	1.28	1.22	1.10	0.80	10,734
60804	0.47	0.45	0.53	0.58	0.59	0.60	0.64	0.58	19.84	84,573
60827	0.45	0.43	0.48	0.50	0.49	0.50	0.54	0.51	18.16	27,946

### Traffic

Traffic volume is captured by loop detectors on the Chicago expressway network. These data are collected by the Gateway Traveler Information System and provided by the Illinois Department of Transportation (IDOT). For this study, 211 loop detectors across Chicago from the Kennedy (I-90/94), Eden (I-94), Eisenhower (I-290), Stevenson (I-55), Dan Ryan (I-90/94), Bishop Ford (I-94), and I-57 expressways are used. Each loop detector includes the number of cars that pass a point in the last 5 min. Standard data cleaning processes were applied to remove missing and erroneous data points that may originate from detector malfunction, pavement condition, or from any other reason. Finally, we aggregated traffic volumes to 30-min time periods to be able to merge the traffic dataset with the electricity consumption dataset. Table 2 shows the average traffic volume per lane per 5 min in each zip code across Chicago. Similar to Table 1, we added the area and population for each zip code. In this study, we only focus on 28 zip codes (out of 56 in Chicago) because the expressway system only cross 28 zip codes.

**Table 2:** Average traffic volume (vehicles per lane per 5 min).

Zip code	12 AM to 3 AM	3 AM to 6 AM	6 AM to 9 AM	9 AM to 12 PM	12 PM to 3 PM	3 PM to 6 PM	6 PM to 9 PM	9 PM to 12 AM	Area (km^2^)	Population
60607	20.32	20.97	43.89	44.20	44.65	42.37	42.66	39.62	6.06	30,306
60608	18.68	19.68	39.52	39.95	39.71	34.47	37.27	33.78	16.78	80,011
60609	18.15	19.54	40.87	42.86	47.02	41.59	39.89	35.95	20.18	60,551
60612	16.42	15.00	39.66	41.16	44.75	40.63	41.51	34.72	9.63	32,240
60614	22.42	22.10	43.20	53.33	54.79	46.30	48.54	42.88	10.00	72,391
60616	12.26	12.92	26.62	27.51	28.94	27.07	25.61	22.37	11.58	52,557
60618	13.68	15.46	32.49	35.05	38.01	34.61	35.17	29.71	13.11	94,646
60619	14.05	25.55	51.39	50.65	49.50	50.56	44.02	32.73	15.85	61,372
60620	18.61	19.11	43.09	45.61	48.40	47.05	43.99	37.78	18.13	68,761
60621	16.64	21.46	47.54	46.32	48.12	44.76	41.87	35.73	9.71	26,736
60623	20.71	21.47	42.82	43.25	47.07	45.43	47.08	37.84	14.14	77,732
60624	16.97	16.57	43.00	46.05	48.44	43.93	46.10	37.70	9.19	35,054
60625	14.80	14.69	41.18	48.03	51.21	49.72	44.90	34.15	9.84	78,820
60628	11.74	9.78	18.98	23.95	27.50	29.16	27.70	22.04	28.62	65,008
60630	16.74	18.72	39.09	44.45	46.00	41.39	41.16	36.40	12.28	55,692
60631	17.44	24.40	48.47	53.64	55.24	51.92	48.78	39.36	9.71	28,864
60632	16.60	21.48	42.88	41.39	43.04	45.05	42.88	32.50	19.42	86,715
60633	10.40	9.35	18.12	21.30	24.09	27.33	25.02	19.07	30.10	12,720
60637	22.51	15.16	39.94	44.95	50.27	39.65	42.16	42.02	11.97	46,621
60641	12.53	15.35	27.37	31.38	33.59	28.69	32.07	27.91	10.44	70,163
60642	16.92	17.39	36.33	39.14	37.31	35.46	38.64	33.75	4.53	20,191
60643	12.63	12.05	26.06	30.23	34.43	39.42	34.14	24.22	19.09	48,572
60644	16.25	17.75	38.98	45.32	46.45	42.77	45.15	36.64	9.12	45,919
60647	14.43	15.84	33.13	36.12	37.65	34.36	35.04	30.74	10.33	85,658
60656	20.44	24.41	47.95	54.78	56.17	54.05	51.49	44.28	8.39	28,982
60661	16.33	18.07	40.17	40.77	36.15	36.86	40.52	35.08	0.80	10,734
60804	15.58	21.48	38.39	40.01	44.50	47.07	44.23	31.76	19.84	84,573
60827	13.56	14.91	32.76	34.04	36.33	42.42	36.72	25.27	18.16	27,946

## Results

### Correlation between electricity consumption and traffic volume

First, we can look at the correlation between two time-series datasets, traffic volume and electricity consumption. For that, we utilize the Pearson *r* correlation coefficient to measure the linear relation between electricity consumption and traffic volume. Specifically, we calculate the Pearson coefficients in three levels: loop detector, zip code, and citywide.

**Figure 1:**
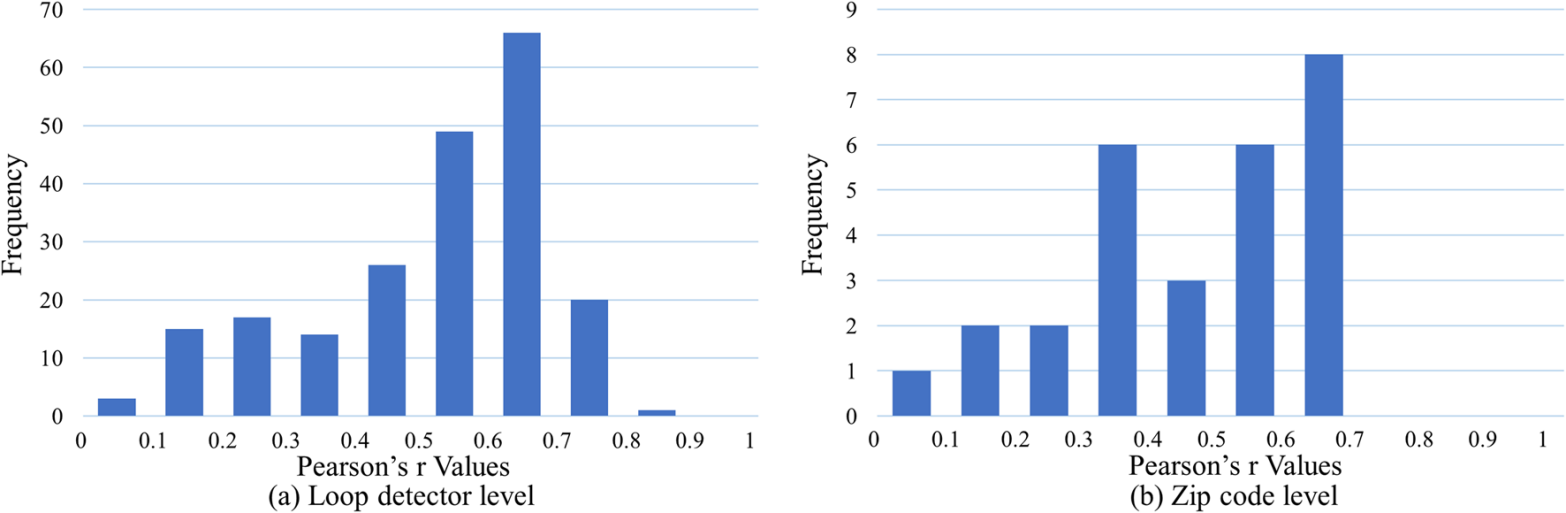
Pearson *r* values.

**Figure 2:**
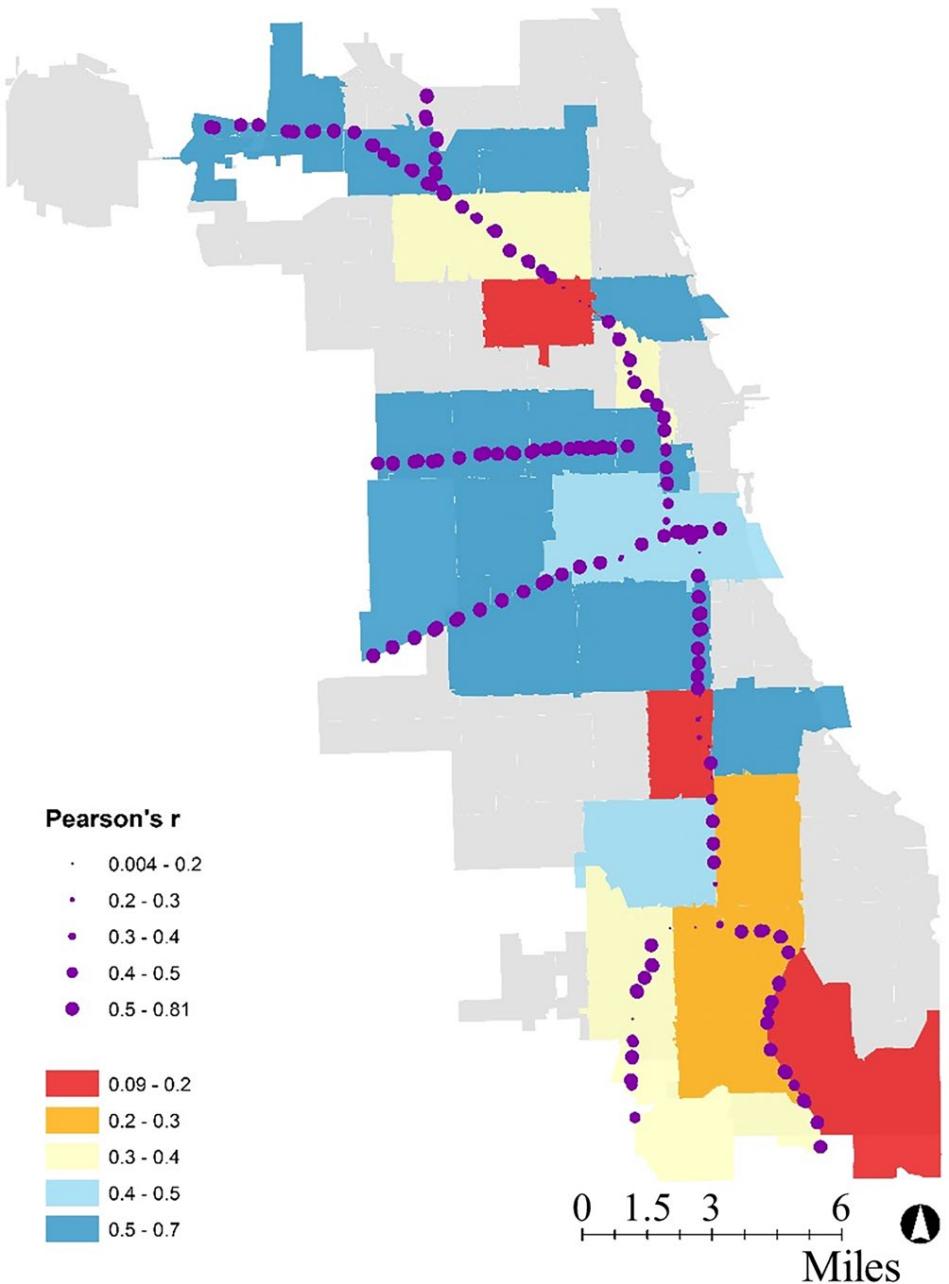
Pearson's r values—Chicago map. Environmental Systems Research Institute (Esri) ArcGIS Desktop 10.8.1 commercial versions were used to perform preliminary data preparation and convert tabular data to spatial data. URL: https://www.esri.com/en-us/arcgis/products/arcgis-desktop/resources.

For each loop detector, we assign the zip code in which the loop detector is located. Then, we calculate the Pearson *r* value for each loop detector across the city. Figure 1a shows the histogram of the Pearson *r* values. They are distributed between 0.004 and 0.81. The highest frequency of Pearson *r* values (66 out of total 211 loop detectors) is in the range [0.6, 0.7). To interpret properly the Pearson results we need to consider where zip codes with similar Pearson *r* values are located. Figure 2 shows the Pearson *r* values of the loop detectors and zip codes on a Chicago map. We can see that most loop detectors and zip codes with similar Pearson *r* values are located near one another.

At the zip code level, we consider all loop detectors in one zip code and calculate the Pearson *r* values for electricity consumption and traffic volume. Figure 1b shows that Pearson *r* values are distributed between 0.09 and 0.66 with eight values being in the range [0.6, 0.7). Figure 2 shows how different zip codes have different correlations between electricity consumption and traffic volume. Except for a few zip codes, the figure suggests that the correlation is higher in the north side and the center of the city, and it decreases as we move south. This difference likely stems from the fact that expressways are used as the boundary between zip codes in the south. On a map, while individual loop detectors belong to one zip code, the drivers getting off the expressway may be going to the adjoining zip code. The low accuracy values therefore do not necessarily suggest the absence of interrelationships, but the lack adequate data.

At the citywide level, we use all the traffic volume data and the corresponding electricity consumption of the zip codes to calculate the overall Pearson *r* value for Chicago that comes to 0.14. Next, we consider a delay in the datasets since a person leaving a building can take time before reaching an expressway and vice versa. Specifically, we increase the delay from 30 min to one day in 30-miniute increments (i.e., 30 min, 60 min, 90 min, …, 1 day) and calculate the correlation of the electricity consumption with the delayed traffic volume. The result of the overall Pearson coefficient correlation shows that the 60 min delay has the highest Pearson value with 0.16, which is low and does not suggest strong correlations at the citywide level.

### Temporal relationships

The goal of this section is to investigate the temporal relationships between traffic volume and electricity consumption. For that, we train LSTM models using traffic volume to predict electricity consumption in a zip code. The first question that arises is the size of the time window that should be used. For example, if we want to predict electricity consumption of a zip code at 4:00 PM, is using traffic volume at 4:00 PM in nearby loop detectors sufficient? Or is it better to consider two time windows with traffic volumes at 3:30 PM and 4:00 PM together to predict the electricity consumption at 4:00 PM? Or is it better to consider more time windows, like 16 from 8:30 AM to 4:00 PM?

To answer this question, we test many time windows for each zip code and compare the performance of the model. Specifically, to predict electricity consumption at time *t*, first we use traffic volume at time *t* and train and assess the performance of the trained LSTM model. Then we use traffic volumes at times *t* and *t* −  30 min and perform the same analysis. The same procedure is repeated until 24 30-min periods are tested, representing a 12-h time window.

**Figure 3:**
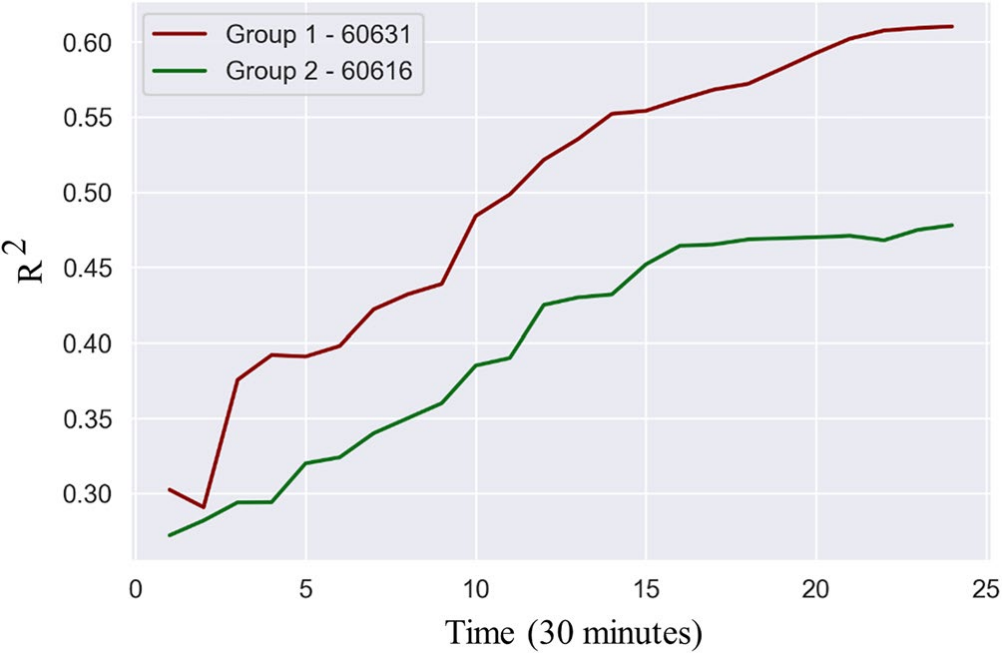
Temporal interrelationships.

The results can be categorized into two groups. In group 1, increasing the time window steadily increases the model performance; Fig. 3 shows an example for zip code 60631. In group 2, increasing the time window initially increases the model performance, but only up to a point (around 16 time periods or 8 h); Fig. 3 shows an example for zip code 60616.

While every zip code has a specific optimal time window, a time window of 16 periods (8 h) tends to perform well across all zip codes since it shows both a high performance for groups 1 and 2 zip codes.

These results are interesting and suggest that the temporal interrelationships between electricity use and travel demand are complex. In particular, we expected the optimal time window to be around 2–3 h for every zip code, to take into account typical rush hour periods, but we find that accuracy keeps increasing until at least 8 h. This means that to predict electricity consumption at 5PM, the use of traffic data between 9AM and 5PM is preferred. We posit that a larger time window of 8 h better captures lifestyle elements, such as an 8-h workday, but this value could vary across by culture.

### Spatial relationships

The goal of this section is to investigate the impact of the distance between zip codes and loop detectors on the relationships between electricity consumption and traffic volume. Therefore, in this section, first, we train LSTM models to predict electricity consumption based on the traffic data from the closest loop detectors, then we increase the distance between loop detectors and the zip code. Here, we use an 8-h time window in our LSTM models (as found preferable in the previous section). To choose the zip codes to study the spatial relationships, we consider four conditions to control the impact of traffic volume from one expressway on the electricity consumption of a zip code. First the zip code should be crossed by only one expressway. Second, there should be only the loop detectors from the same expressway and no other loop detectors from other expressways in a radius of 5 km to limit the amount of noise fed to the model. Third, the zip code should be far enough from the boundaries of Chicago so we can have loop detectors on both side of the zip codes. Fourth, the accuracy of the LSTM model should be significantly more than zero to suggest the existence of a relationship. We applied these four conditions on the Chicago map and few zip codes satisfied them. As an example, we select three zip codes to study the relationships between electricity and travel demand.

To investigate the spatial relationship, we select one set of loop detectors that cross the zip code; each set has one loop detector in one direction of the expressway (toward the zip code) and one in the other direction (away from the zip code). The initial set is the ones closest to the centroid of the zip code. Then, we increase the distance and consider two new loop detectors further away from the centroid of the zip code. The procedure is repeated several times to loop detectors further away on the same expressway. Each time, a model is trained and the performance is compared.

**Figure 4:**
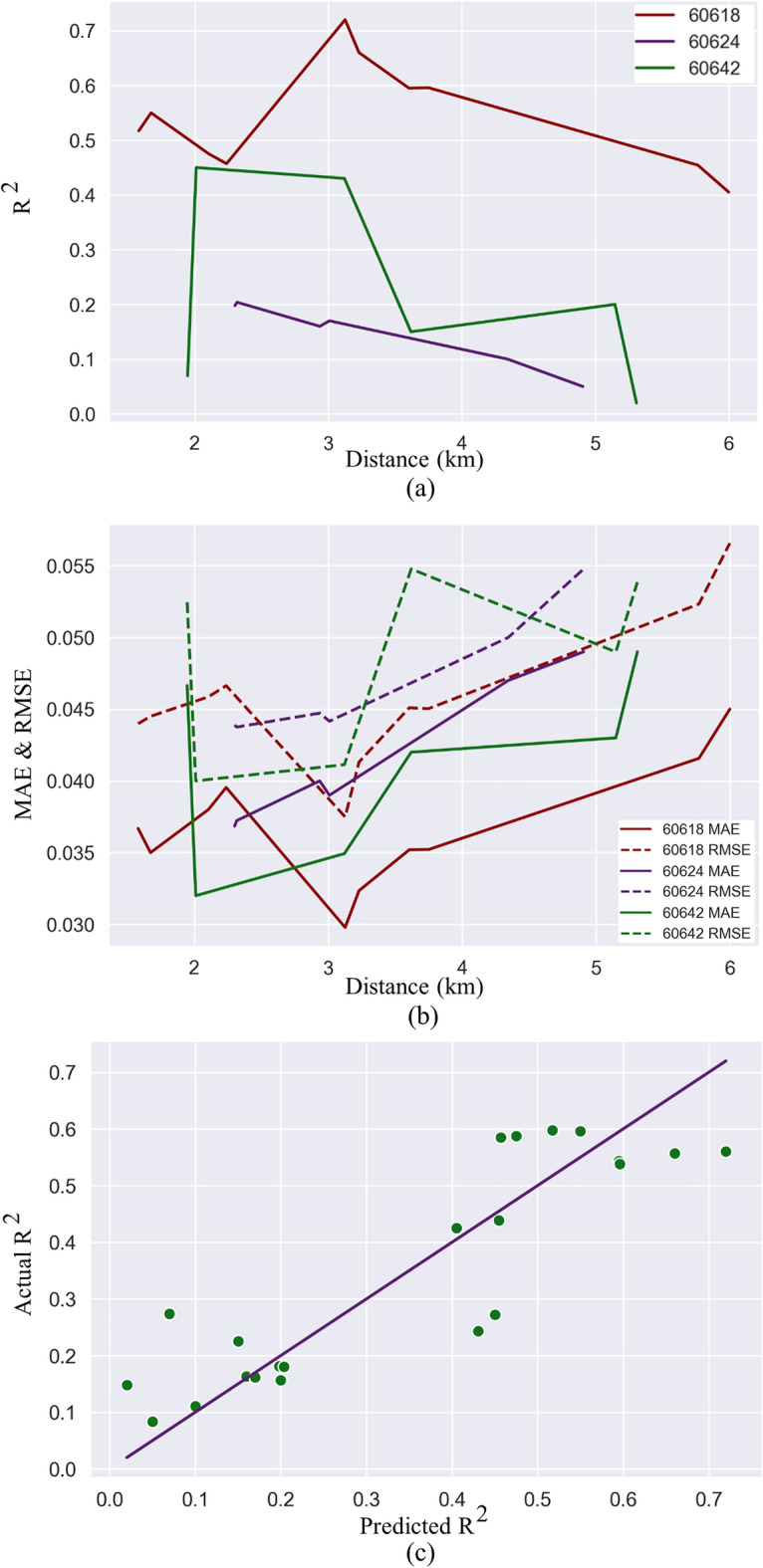
Spatial relationships between electricity consumption and travel demand.

Figure 4 shows the accuracy and errors of the models in terms of R^2^, MAE, and RMSE. In Fig. 4a,b, the average distance between the set of loop detectors (one for each direction) and the centroid of the corresponding zone is shown on the x-axis.

The purple line in Fig. 4a shows the spatial relationship found in zip code 60624. Here, increasing the distance between loop detectors and zip code reduces the accuracy and increases the MAE and RMSE. As expected, increasing the distance reduces the relationships between electricity consumption and traffic volume in this case.

Second, the dark red line in Fig. 4a is for zip code 60618. There, we observe that by increasing the distance, the model accuracy first increases and then it decreases after a certain distance. This phenomenon was unexpected since it suggests that loop detectors located in other zip codes are better able to predict electricity consumption. To further analyze the spatial relationships, we can use all loop detectors in the same zip code to predict the electricity consumption, which we present in the next section.

Finally, the green line in Fig. 4a shows the third type of spatial relationship. Here, increasing distance has no straightforward impact on the model performance.

Overall, we find that complex and unobvious relationships can exist between electricity consumption and traffic volume. Nonetheless, we should consider that each zip code has its own attributes, and to capture these attributes we can include a zip-code level fixed effect as is common in econometrics. Fixed effect variables are used to capture unique features of a data point despite the presence of common attributes^63^. What we can do here is to express R^2^ values as a function of distance from the zip code centroid. But because electricity consumption is collected at the zip code level—a surface area in square kilometers—we should use the square of the distance in our model instead. Our model therefore becomes:

1 $${RSquared}_{ij}={a}_{0}+{a}_{1}\times {distance}_{ij}^{2}+{zc}_{j}+{\varepsilon }_{ij}$$

where *RSquared*_*ij*_ is the accuracy of model *i* in zip code *j*, ${distance}_{ij}$ is the distance between loop detectors and the zip code centroid in the model *i* in zip code *j*, ${zc}_{j}$ is the zip code fixed effect to distinguish between zip codes, ${\varepsilon }_{ij}$ is the error term, and ${a}_{0}$ is the constant term.

The result of the regression is as follows ${a}_{0}=0.293$ and ${a}_{1}=-0.0052$ with a p-value of 0.04, and the zip code fixed effect values are 0.317 for zip code 60618 and − 0.085 for 60624 (note that since we have three zip codes, we have two fixed effect values for the zip codes). The R^2^ of the general fit is 0.78. Figure 4c shows the actual versus predicted values of R^2^ using Eq. (1) and the coefficient values that we calculated. We find a negative relationship with the value of 0.0052 between distance squared and R^2^ values. In other words, we find that increasing the squared distance by one square kilometer generally decreases the accuracy of the model by 0.0052. An ANOVA test is also performed to test the null hypothesis (i.e., whether all variables could be statistically zero). Table 3 shows the result of the ANOVA test. Because the value of the F statistic is 21.77, which is greater than F(3, 18) = 2.416, the null hypothesis can be rejected with a 99% confidence level.

**Table 3:** ANOVA test results.

	Df	Sum Sq	Mean Sq	F value	Pr(> F)
Model	3	0.77939	0.25979	21.77	< 0.00009***
Residuals	18	0.21483	0.01193		
Total	21	0.99422	0.04734		

Significance Level: ‘***’ 0.001.

Overall, despite a careful selection of zip codes, we can see that the spatial relationship between traffic volume and electricity consumption are also complex, but they exist. More work is needed to gain a better understanding of these relationships.

### Prediction models across the city

Here, we train two sets of models. The first set of models uses all loop detectors in a zip code to predict the electricity consumption of the zip code. The second set of models uses single loop detectors to predict electricity consumption of the zip code in which they are located.

In the first set of models, we develop 28 LSTM models for the 28 zip codes that are crossed by at least one expressway in Chicago. The input of the models is 8 h of traffic volume collected by all loop detectors in a zip code (8 h is selected since it performed well across all zip codes in the temporal interrelationships section). The output is the average electricity consumption of the zip code at the end of the 8-h period.

**Figure 5:**
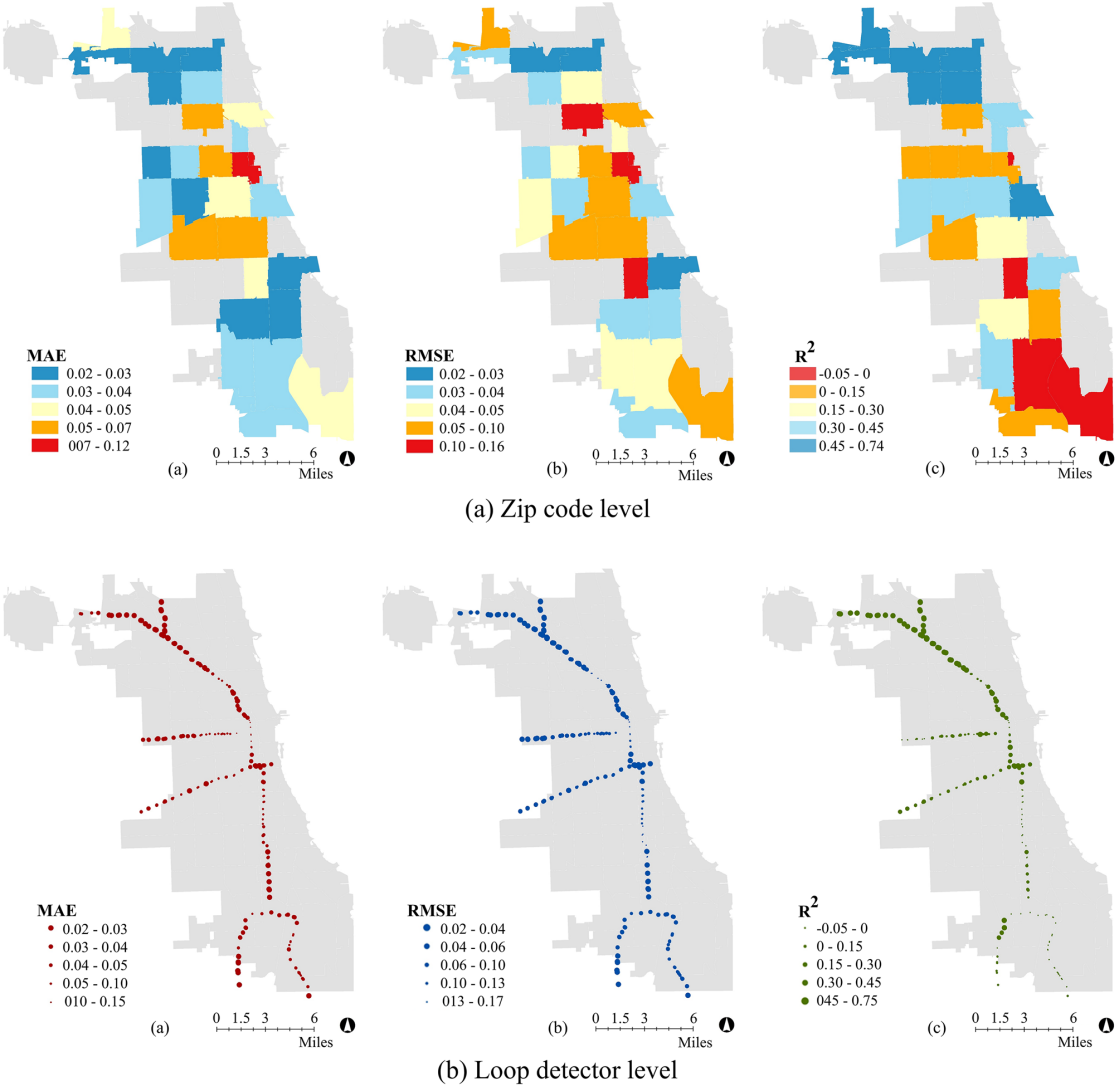
Performance of zip code and loop detector level models that use traffic volume to predict electricity consumption. Environmental Systems Research Institute (Esri) ArcGIS Desktop 10.8.1 commercial versions were used to perform preliminary data preparation and convert tabular data to spatial data. URL: https://www.esri.com/en-us/arcgis/products/arcgis-desktop/resources.

Figure 5a shows maps of Chicago with the R^2^, MAE, and RMSE values of the 28 LSTM models. First, we can see the overall performance of the models are better in the north side of Chicago than the south side. As mentioned above, one problem we face with the south side of Chicago is that the expressway serves as a boundary between zip codes. It is therefore more difficult to determine whether drivers exiting the expressway stay in the zip code where the loop detector is located or whether they go to the adjoining zip code.

In the second set of models, we train 211 LSTM models for the 211 loop detectors in Chicago to predict the electricity consumption of the zip code to which each loop detector belongs. For each model, R^2^, MAE, and RMSE are calculated and shown in Fig. 5b; larger circles represent higher accuracies. We can see that accuracies are higher in the north side, similar to the previous models, likely again because the expressways serve as a boundary between zip codes in the south.

Overall, these results suggest that electricity demand and travel demand are interrelated, as in, one is related to the other and vice versa, but these interrelationships can be complex.

Interestingly, we note that the model performances are similar whether all or single loop detector are selected. This result suggest that single loop detectors may be sufficient to capture relationships between travel demand and electricity consumption. Another future area of research could focus on how much data is needed to capture interrelationships between infrastructure systems.

## Discussion

The results show that the correlation between electricity consumption and traffic volume is complex since it varies by zip code across Chicago with Pearson values ranging between 0.04 and 0.81. Second, the optimum time window to analyze the temporal interrelationship between electricity consumption and traffic volume is 8 h. Furthermore, we investigated the spatial relationship between electricity consumption and travel demand. Despite finding complex and unobvious relationships, we detected a global linear relationship between distance squared and R^2^ values; specifically, that increasing the squared distance by one square kilometer decreases the accuracy of the model by 0.0052. Finally, we developed 239 LSTM models to predict electricity consumption of a zip code using traffic volume from the same zip code and found a range of model performance across the city.

Overall, the idea of the study is novel. The articles listed in the literature review section explore various methods for short-term load forecasting and related applications in the field of energy and transport. While they also discuss applications of these methods in energy management, travel mode choice modeling, and accident detection, none of them explore the spatial relationship between electricity consumption and travel demand. As our study is novel, it cannot be compared with other articles. Nevertheless, we recognize that the interrelationships between traffic volume and electricity consumption are likely influenced by a range of complex and context-specific factors from obvious factors like the presence of alternate travel modes (e.g., transit, walk, bike) to less obvious factors related to household characterisitcs^13^, and daily^10^ and seasonal^12^ effects.

Furthermore, this study has several limitations. In particular, it would have benefited from having access to origin–destination data (not for a typical date but for a specific day to compare energy use patterns) and to more detailed travel volume data (beyond traffic volumes on the expressway system).

In terms of policy implications, this work suggests that policies made to impact one infrastructure system can impact others. For example, many cities have adopted time-varying pricing practices for tollways (e.g., Singapore) and public transport (e.g., Washington DC) to encourage people to avoid rush hour periods and lessen congestion, which must have an impact of electricity consumption (as well as other resources such as water and gas). With the global push toward infrastructure decentralization and distribution^65^, we recommend better coordination among utilities and transport service providers.

Future work should focus on further understanding these interrelationships, ideally using other more spatially disaggregate datasets. It is aligned with limitations from other research^25^, which uses a similar methodology and mentions that the exploration of many datasets from distant energy contexts is necessary for a broader understanding of the problem. Another future area of research is on how much data is needed to capture interrelationships between infrastructure systems. For instance, increasing the spatial resolution of the data by collecting information at a more disaggregated level, as well as incorporating data on public transportation usage and other mobility-related variables, could further provide additional insights and improve the accuracy of models.

## Material and methods

### Long short-term memory (LSTM)

**Figure 6:**
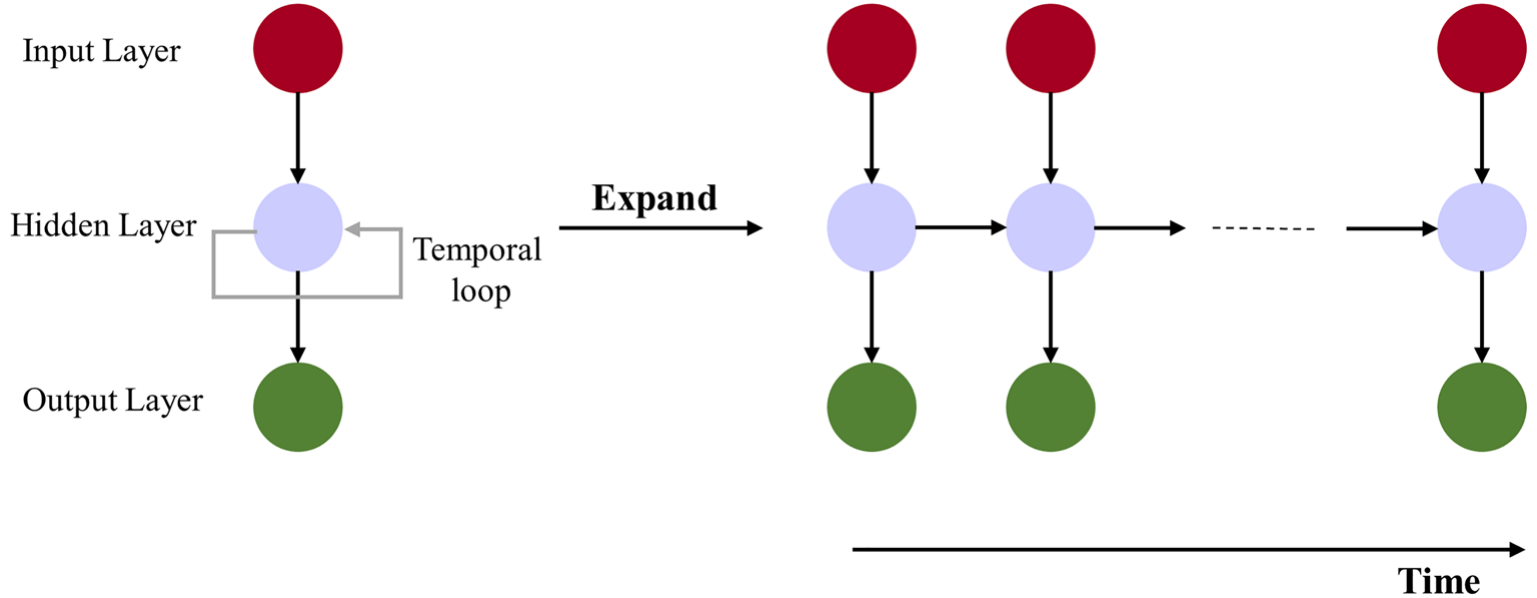
Structure of RNN model. Microsoft Visio 2019 was used to draw the visual concept of LSTM based on the Authors’ understanding of the model. https://www.microsoft.com/en-us/microsoft-365/visio/flowchart-software.

Neural Networks (NN) are one of the most widely used types of machine learning techniques. They are made of three layers: (a) input, (b) output, and (c) hidden. The most common types of NN have a cost function, and the goal is to minimize this cost function through re-adjusting the weights (i.e., model parameters) using a backpropagation technique. Recurrent Neural Networks (RNN) are more advanced and complex models that belong to the family of deep learning techniques. In RNN, a temporal loop connects the hidden layer to itself, meaning that the hidden layer not only impacts the output but also gives feedback to itself. The structure of an RNN model is shown in Fig. 6.

In sequence prediction problems, Long Short-Term Memory (LSTM) networks are a specific type of RNN that can learn the dependency in the sequence of time-series data. Since there could be a lag between the events of interest in a time series, these networks can perform well with different types of problem such as classification, processing, and prediction using time series data. One important issue in the standard RNN models is the inefficiency of the model to learn when there are time lags greater than five to ten discrete time steps between the input data target variable that can cause a vanishing gradient—that is, the gradient is too small, preventing the weight from changing its value. LSTMs were developed to cope with the problem of vanishing gradient. They can learn to connect minimal time lags when there exist many discrete time steps by enforcing constant error flow through special units, called cells; see Eqs. (2) and (3). In LSTMs, the flow of information is controlled through gates that keep or override information in the memory cell, forgetting previous information, and deciding how to access memory cell; see Eq. (4). An LSTM consists of three gates. The two gates that learn to open and close access to error within the memory cell are input and output gates. The third type of gate is the forget gate that has a specific role to reset operations for the cells. In another word, the input gate decides how much of the new state *h*[*t*] should be updated; the output gate determines the portion of the state that must be outputted; and the forget gate decides the part of the information that needs to be forgotten and eliminated from the previous cell state *h*[*t*-1]. The main flow of information happens through a cell state. The cell state is updated in a forward process and the output is computed as displayed in Eq. (5):

2 $$\text{forget gate}:{\upsigma }_{f}\left[t\right]=\upsigma ({\text{W}}_{f}\cdot x\left[t\right] +{\text{R}}_{f}\cdot \text{y}\left[t-1\right] + {\text{b}}_{f})$$

3 $${\text{candidate state}}:\tilde{h}\left[ t \right] = {\text{g}}_{1} \left( {{\text{W}}_{h} \cdot x\left[ t \right]{ } + {\text{R}}_{h} \cdot {\text{y}}\left[ {t - 1} \right]{ } + {\text{ b}}_{h} } \right)$$

4 $$\text{input gate}:{\upsigma }_{u}\left[t\right]=\upsigma ({\text{W}}_{u}\cdot x\left[t\right] +{\text{R}}_{u}\cdot \text{y}\left[t-1\right] + {\text{b}}_{u})$$

5 $${\text{cell state}}:{\text{h}}\left[ t \right] = {\upsigma }_{u} \left[ t \right] \left( \cdot \right) \tilde{h}\left[ t \right] + {\upsigma }_{f} \left[ t \right] \left( \cdot \right) {\text{h}}\left[ {t - 1} \right]$$

6 $$\text{output gate}:{\upsigma }_{o}\left[t\right]=\upsigma ({\text{W}}_{o}\cdot x\left[t\right] +{\text{R}}_{o}\cdot \text{y}\left[t-1\right] + {\text{b}}_{o})$$

7 $$\text{output}:\text{y}\left[t\right]= {\upsigma }_{o}\left[t\right] \left(\cdot \right) {\text{g}}_{2}(\text{h}\left[t\right])$$

where *x*[*t*] is the input at time *t*, *σ*(·) is a sigmoid function, *g*_*1*_(·) and *g*_*2*_(·) denote the point wise nonlinear activation function, (∙) denotes the entry wise multiplication between two vectors, *R*_*o*_, *R*_*u*_, *R*_*h*_, and *R*_*f*_ represents weight matrices of the recurrent connections, *W*_*o*_, *W*_*u*_, *W*_*h*_, and *W*_*f*_ are weight matrices for the inputs of LSTM cells, *b*_*o*_, *b*_*u*_, *b*_*f*_, and *b*_*h*_ are bias vectors^5^. The LSTM model was developed in Python (v3.7.3) using the Keras (v2.2.4) Deep Learning Library that itself uses TensorFlow (v2.0.0b0) in the backend.

### Model execution and validation

In this study, the dataset is split into two groups: the first 22 days of November for training and the last 8 days of November for testing. The groups were not split randomly on purpose to ensure both the training and testing sets had weekdays and weekends. Moreover, it is a common practice when modeling time series to use earlier data for training and later data for testing. The premise is that a good model should be able to capture new, unseen trends. We kept the same practice even our goal is not to develop the best performing model, but to study interrelationships.

Around 250 LSTM models were trained and compared to select optimal hyperparameters. The hyperparameters used in the end are as follows: number of epochs: 200; batch size: 50; learning rate: 0.001, optimizer: Adam; activation function: sigmoid; loss function: Binary crossentropy.

In terms of performance, we use goodness of fit ${R}^{2}$, mean absolute error (MAE), and root mean squared error (RMSE) defined as:

8 $${R}^{2}=1-\frac{\sum_{i}{\left({y}_{i}-{\widehat{y}}_{i}\right)}^{2}}{\sum_{i}{\left({y}_{i}-\overline{y }\right)}^{2}}$$

9 $$MAE=\frac{\sum_{i=1}^{n}|{y}_{i}-{\widehat{y}}_{i}|}{n}$$

10 $$RMSE=\sqrt{\frac{1}{n}{\sum }_{i=1}^{n}{({y}_{i}-{\widehat{y}}_{i})}^{2}}$$

where ${y}_{i}$ is the actual value of a data point, ${\widehat{y}}_{i}$ is the predicted value,$n$ is the number of data points, and $\overline{y }$ is the mean value of all $n$ actual values.

To calculate the correlation between two time-series we use Pearson *r* value, defined as:

11 $$r= \frac{\sum (x-{m}_{x})(y-{m}_{y})}{\sqrt{\sum {(x-{m}_{x})}^{2}\sum {(y-{m}_{y})}^{2}}}$$

where $x$ and $y$ are the data points of two time-series and ${m}_{x}$ and ${m}_{y}$ are the mean of the vector $x$ and $y$ respectively.

## Data Availability

Traffic volume data is collected by the Gateway Traveler Information System and provided by the Illinois Department of Transportation (IDOT) to some of the team members. The authors were not granted the right to share the data. Electricity data was collected from Commonwealth Edison (ComEd). Anyone can access it for a fee at https://www.comed.com/SmartEnergy/InnovationTechnology/pages/anonymousdataservice.aspx (accessed March 15, 2023).

## References

[1] Derrible S (2019). Urban engineering for sustainability.

[2] Sarwat AI, Sundararajan A, Parvez I, Moghaddami M, Moghadasi A (2018). Toward a smart city of interdependent critical infrastructure networks. Sustainable interdependent networks.

[3] Movahedi, A, & Derrible, S. Interrelationships between electricity, gas, and water consumption in large‐scale buildings. *J. Ind. Ecol.* 1–16. 10.1111/jiec.13097 (2020).

[4] Zhang P, Zhen (Sean) Qian. (2018). User-centric interdependent urban systems: using time-of-day electricity usage data to predict morning roadway congestion. Transport. Res. Part C Emerg. Technol..

[5] Marvin S, Slater S (1997). The new urban infrastructure crisis competition for urban space. Public Works Manag. Policy.

[6] Fan, Y., Lee, A., Parker, N., Scheitrum, D., Dominguez-Faus, R., Jaffe, A. M., & Medlock III, K. Geospatial, temporal and economic analysis of alternative fuel infrastructure: The case of freight and US natural gas markets.* Energy J.* **38**(6) (2017).

[7] Hunt, S. D. A general theory of competition: Resources, competences, productivity, economic growth. *Sage Publications *(1999).

[8] Ahmad N, Derrible S (2018). An information theory based robustness analysis of energy mix in US States. Energy Policy.

[9] Bikcora C, Verheijen L, Weiland S (2018). Density forecasting of daily electricity demand with ARMA-GARCH, CAViaR, and CARE econometric models. Sustain. Energy Grids Netw..

[10] Wu F, Cattani C, Song W, Zio E (2020). Fractional ARIMA with an improved cuckoo search optimization for the efficient Short-term power load forecasting. Alex. Eng. J..

[11] Torkzadeh R., Mirzaei, A., Mirjalili, M. M., Anaraki, A. S., Sehhati, M. R., & Behdad, F. Medium term load forecasting in distribution systems based on multilinear regression & principal component analysis: A novel approach. in* Proc. 19th Conf. Elect. Power Distrib. Netw. (EPDC)*, May 2014, pp. 66–70 (2014).

[12] Wang ZX, Li Q, Pei LL (2018). A seasonal GM (1, 1) model for forecasting the electricity consumption of the primary economic sectors. Energy.

[13] Zheng Z, Chen H, Luo X (2019). A Kalman filter-based bottom-up approach for household short-term load forecast. Appl. Energy.

[14] Dong Y, Ma X, Fu T (2021). Electrical load forecasting: A deep learning approach based on K-nearest neighbors. Appl. Soft Comput..

[15] Esener İI, Yüksel T, Kurban M (2015). Short-term load forecasting without meteorological data using AI-based structures. Turk. J. Electr. Eng. Comput. Sci..

[16] Zor, K., Timur, O., & Teke, A. A state-of-the-art review of artificial intelligence techniques for short-term electric load forecasting. In *2017 6th international youth conference on energy (IYCE)* (pp. 1–7). IEEE (2017).

[17] Lee D, Derrible S, Pereira FC (2018). Comparison of four types of artificial neural network and a multinomial logit model for travel mode choice modeling. Transp. Res. Rec..

[18] Seyrfar A, Ataei H, Movahedi A, Derrible S (2021). Data-driven approach for evaluating the energy efficiency in multifamily residential buildings. Pract. Period. Struct. Des. Constr..

[19] Kashani H, Movahedi A, Morshedi MA (2019). An agent-based simulation model to evaluate the response to seismic retrofit promotion policies. Int. J. Disaster Risk Reduct..

[20] Liao GC, Tsao TP (2006). Application of a fuzzy neural network combined with a chaos genetic algorithm and simulated annealing to short-term load forecasting. IEEE Trans. Evol. Comput..

[21] Ling SH, Leung FHF, Lam HK, Lee YS, Tam PKS (2003). A novel genetic-algorithm-based neural network for short-term load forecasting. IEEE Trans. Industr. Electron..

[22] Han XS, Han L, Gooi HB, Pan ZY (2012). Ultra-short-term multi-node load forecasting—a composite approach. IET Gener. Transm. Distrib..

[23] Parsa, A.B., Movahedi, A., Taghipour, H., Derrible, S., & Mohammadian, A. Toward safer highways, application of XGBoost and SHAP for real-time accident detection and feature analysis, *Accid. Anal. Prev.,* **136** (2020). 10.1016/j.aap.2019.105405 10.1016/j.aap.2019.105405 31864931

[24] Badhrudeen, M., Naranjo, N., Movahedi, A., & Derrible, S. Machine learning based tool for identifying errors in CAD to GIS converted data. In Proc., *CIGOS 2019, Innovation for Sustainable Infrastructure,* 1185–1190. Singapore: Springer (2020).

[25] Lee D, Mulrow J, Haboucha CJ, Derrible S, Shiftan Y (2019). Attitudes on autonomous vehicle adoption using interpretable gradient boosting machine. Transp. Res. Rec..

[26] Bouktif S, Fiaz A, Ouni A, Serhani MA (2018). Optimal deep learning lstm model for electric load forecasting using feature selection and genetic algorithm: Comparison with machine learning approaches. Energies.

[27] Ren Y, Suganthan PN, Srikanth N, Amaratunga G (2016). Random vector functional link network for short-term electricity load demand forecasting. Inf. Sci..

[28] Guo Z, Zhou K, Zhang X, Yang S (2018). A deep learning model for short-term power load and probability density forecasting. Energy.

[29] Sarvestani SE, Hatam N, Seif M, Kasraian L, Lari FS, Bayati M (2022). Forecasting blood demand for different blood groups in Shiraz using auto regressive integrated moving average (ARIMA) and artificial neural network (ANN) and a hybrid approaches. Sci. Rep..

[30] Ward T, Johnsen A, Ng S (2022). Forecasting SARS-CoV-2 transmission and clinical risk at small spatial scales by the application of machine learning architectures to syndromic surveillance data. Nat. Mach. Intell..

[31] Ma R, Zheng X, Wang P (2021). The prediction and analysis of COVID-19 epidemic trend by combining LSTM and Markov method. Sci. Rep..

[32] Benvenuto, D., Giovanetti, M., Vassallo, L., Angeletti, S., & Ciccozzi, M. Application of the ARIMA model on the COVID-2019 epidemic dataset. Data Brief. **29**, 105340 (2020). 10.1016/j.dib.2020.105340. 10.1016/j.dib.2020.105340 PMC7063124 32181302

[33] Abduljabbar RL, Dia H, Tsai PW (2021). Development and evaluation of bidirectional LSTM freeway traffic forecasting models using simulation data. Sci. Rep..

[34] Wang W, Zhang H, Li T, Guo J, Huang W, Wei Y, Cao J (2020). An interpretable model for short term traffic flow prediction. Math. Comput. Simul..

[35] Hor CL, Watson SJ, Majithia S (2005). Analyzing the impact of weather variables on monthly electricity demand. IEEE Trans. Power Syst..

[36] Apadula F, Bassini A, Elli A, Scapin S (2012). Relationships between meteorological variables and monthly electricity demand. Appl. Energy.

[37] Quan SJ, Economou A, Grasl T, Yang PPJ (2020). An exploration of the relationship between density and building energy performance. Urban Des. Int..

[38] Barton, H. City of well-being: A radical guide to planning. Taylor & Francis (2016).

[39] Proque, A. L., dos Santos, G. F., Junior, A. A. B., & Larson, W. D. Effects of land use and transportation policies on the spatial distribution of urban energy consumption in Brazil. *Energy Econ.* 104864 (2020).

[40] Yang PP (2015). Energy resilient urban form: A design perspective. Energy Proc..

[41] Stephan A, Crawford RH (2016). The relationship between house size and life cycle energy demand: Implications for energy efficiency regulations for buildings. Energy.

[42] Estiri H (2016). Household energy consumption and housing choice in the US residential sector. Hous. Policy Debate.

[43] Filippín C, Ricard F, Larsen SF (2013). Evaluation of heating energy consumption patterns in the residential building sector using stepwise selection and multivariate analysis. Energy Build..

[44] Hunt, R., & Suhr, M. Old House Eco Handbook: A practical guide to retrofitting for energy efficiency and sustainability. White Lion Publishing. (2019).

[45] Sanaieian H, Tenpierik M, Van Den Linden K, Seraj FM, Shemrani SMM (2014). Review of the impact of urban block form on thermal performance, solar access and ventilation. Renew. Sustain. Energy Rev..

[46] Ko Y (2013). Urban form and residential energy use: A review of design principles and empirical findings. J. Plan. Lit..

[47] Karatas A, Stoiko A, Menassa CC (2016). Framework for selecting occupancy-focused energy interventions in buildings. Build. Res. Inf..

[48] Broberg T, Egüez A (2018). Blame it on the owner—Ownership and energy performance of multi-dwelling buildings. Energy Econ..

[49] Li, C., Song, Y., Kaza N., & Burghardt R. Explaining spatial variations in residential energy usage intensity in Chicago: The Role of Urban Form and Geomorphometry. *J. Plan. Educ. Res.* 0739456X19873382 (2019).

[50] Estiri H, Zagheni E (2019). Age matters: Ageing and household energy demand in the United States. Energy Res. Soc. Sci..

[51] Umit R, Poortinga W, Jokinen P, Pohjolainen P (2019). The role of income in energy efficiency and curtailment behaviours: Findings from 22 European countries. Energy Res. Soc. Sci..

[52] Derrible S (2018). An approach to designing sustainable urban infrastructure. MRS Energy Sustain..

[53] Cordova J, Sriram LMK, Kocatepe A, Zhou Y, Ozguven EE, Arghandeh R (2018). Combined electricity and traffic short-term load forecasting using bundled causality engine. IEEE Trans. Intell. Transp. Syst..

[54] Madhavi, K. L., Gilanifar, M., Zhou, Y., Ozguven, E. E., & Arghandeh, R. Multivariate deep causal network for time series forecasting in interdependent networks. In *2018 IEEE Conference on Decision and Control (CDC)* (pp. 6476–6481). IEEE (2018).

[55] Gilanifar M, Wang H, Ozguven EE, Zhou Y, Arghandeh R (2019). Bayesian spatiotemporal gaussian process for short-term load forecasting using combined transportation and electricity data. ACM Trans. Cyber-Phys. Syst..

[56] Aparicio, J., Rosca, J., Mediger, M., Essl, A., Arzig, K., & Develder, C. Exploiting road traffic data for very short term load forecasting in smart grids. In *ISGT 2014* (pp. 1–5). IEEE (2014).

[57] ComEd, Anonymous Data Service Product Offering. (2021). https://www.comed.com/SmartEnergy/InnovationTechnology/pages/anonymousdataservice.aspx. Accessed 05 Oct 2021.

[58] Wong, J., & Rajagopal, R. A simple way to use interval data to segment residential customers for energy efficiency and demand response program targeting. *In* ACEEE Proceedings (2012).

[59] Parker, S. A., Hunt, W. D., McMordie Stoughton, K., Boyd, B. K., Fowler, K. M., Koehler, T. M., & Pugh, R. Metering best practices: A guide to achieving utility resource efficiency, release 3.0 (No. PNNL-23892-Rel. 3.0). Pacific Northwest National Lab.( PNNL), Richland, WA (United States) (2015).

[60] Fumo N, Biswas MR (2015). Regression analysis for prediction of residential energy consumption. Renew. Sustain. Energy Rev..

[61] Mashima, D., & Roy, A. Privacy preserving disclosure of authenticated energy usage data. In *2014 IEEE international conference on smart grid communications (SmartGridComm)* (pp. 866–871). IEEE (2014).

[62] Martínez, S., Sebé, F., & Sorge, C. Measuring privacy in smart metering anonymized data (2020). arXiv preprint arXiv:2002.04863.

[63] Dietrich, A., Leibenger, D., & Sorge, C. On the Lack of Anonymity of Anonymized Smart Meter Data: An Empiric Study. In *2020 IEEE 45th Conference on Local Computer Networks (LCN)* (pp. 405–408). IEEE (2020).

[64] Rice K, Higgins JPT, Lumley T (2018). A re-evaluation of fixed effect(s) meta-analysis. J. R. Stat. Soc. A. Stat. Soc..

[65] Helmrich A, Markolf S, Li R, Carvalhaes T, Kim Y, Bondank E, Chester M (2021). Centralization and decentralization for resilient infrastructure and complexity. Environ. Res. Infrastruct. Sustain..

